# Downregulation of SMOC1 is associated with progression of colorectal traditional serrated adenomas

**DOI:** 10.1186/s12876-024-03175-1

**Published:** 2024-03-01

**Authors:** Hironori Aoki, Akira Takasawa, Eiichiro Yamamoto, Takeshi Niinuma, Hiro-o Yamano, Taku Harada, Toshiyuki Kubo, Akira Yorozu, Hiroshi Kitajima, Kazuya Ishiguro, Masahiro Kai, Akio Katanuma, Toshiya Shinohara, Hiroshi Nakase, Tamotsu Sugai, Makoto Osanai, Hiromu Suzuki

**Affiliations:** 1 Department of Molecular Biology, Sapporo Medical University School of Medicine, S1, W17, Chuo-Ku, Sapporo, 060-8556 Japan; 2 Center for Gastroenterology, Teine-Keijinkai Hospital, Sapporo, Japan; 3 Department of Gastroenterology and Endoscopy, Koyukai Shin-Sapporo Hospital, Sapporo, Japan; 4 Department of Pathology, Sapporo Medical University School of Medicine, Sapporo, Japan; 5 Department of Gastroenterology and Hepatology, Sapporo Medical University School of Medicine, Sapporo, Japan; 6 Department of Pathology, Teine-Keijinkai Hospital, Sapporo, Japan; 7 Department of Molecular Diagnostic Pathology, Iwate Medical University, Morioka, Japan

**Keywords:** SMOC1, Colorectal cancer, Serrated lesion, Traditional serrated adenoma

## Abstract

**Background:**

Aberrant DNA methylation is prevalent in colorectal serrated lesions. We previously reported that the CpG island of SMOC1 is frequently methylated in traditional serrated adenomas (TSAs) and colorectal cancers (CRCs) but is rarely methylated in sessile serrated lesions (SSLs). In the present study, we aimed to further characterize the expression of SMOC1 in early colorectal lesions.

**Methods:**

SMOC1 expression was analyzed immunohistochemically in a series of colorectal tumors (*n* = 199) and adjacent normal colonic tissues (*n* = 112).

**Results:**

SMOC1 was abundantly expressed in normal colon and SSLs while it was significantly downregulated in TSAs, advanced adenomas and cancers. Mean immunohistochemistry scores were as follows: normal colon, 24.2; hyperplastic polyp (HP), 18.9; SSL, 23.8; SSL with dysplasia (SSLD)/SSL with early invasive cancer (EIC), 15.8; TSA, 5.4; TSA with high grade dysplasia (HGD)/EIC, 4.7; non-advanced adenoma, 21.4; advanced adenoma, 11.9; EIC, 10.9. Higher levels SMOC1 expression correlated positively with proximal colon locations and flat tumoral morphology, reflecting its abundant expression in SSLs. Among TSAs that contained both flat and protruding components, levels of SMOC1 expression were significantly lower in the protruding components.

**Conclusion:**

Our results suggest that reduced expression of SMOC1 is associated with progression of TSAs and conventional adenomas and that SMOC1 expression may be a biomarker for diagnosis of serrated lesions and risk prediction in colorectal tumors.

**Supplementary Information:**

The online version contains supplementary material available at 10.1186/s12876-024-03175-1.

## Introduction

Colorectal cancers (CRCs) are a heterogeneous family of diseases that develop through several distinct molecular pathways. Most CRCs develop from conventional adenomas or serrated lesions, with the serrated pathway accounting for 15–35% of CRCs [1, 2]. Traditional serrated adenomas (TSAs) are the rarest colorectal serrated lesions [1, 3], accounting for less than 1% of all colorectal polyps and for 1–7% of all serrated lesions [2–4]. The available evidence now suggests that accumulation of molecular alterations drives the development of TSAs. Initially, TSAs present KRAS or BRAF mutations, which activate the mitogen-activated protein kinase (MAPK) pathway [4–7]. Activation of the Wnt pathway via PTPRK-RSPO3 fusion or mutations in RNF43, APC or CTNNB1 are also frequently observed in TSAs [8–10]. In addition to genetic alterations, epigenetic changes, including aberrant DNA methylation, play a major role in the pathogenesis of TSAs. Concurrent hypermethylation of multiple CpG islands, referred to as the CpG island methylator phenotype (CIMP), is preferentially found in TSAs [7, 11]. Positive correlation between BRAF mutations and CIMP-high (CIMP-H) or KRAS mutations and CIMP-low (CIMP-L) are well documented in TSAs, suggesting TSAs may develop through multiple molecular pathways in which both genetic and epigenetic alterations drive tumorigenesis [7, 11].

In an earlier study, we screened for aberrant DNA methylation that could contribute to the development of TSAs. We observed that SMOC1 (SPARC-related molecular calcium-binding 1) was frequently methylated and silenced in TSAs as well as in advanced adenomas and CRCs [12]. We also found that SMOC1 methylation correlated with KRAS mutation and CIMP-L in TSAs. Ectopic expression of SMOC1 suppressed CRC cell proliferation, suggesting its tumor suppressor function. In contrast to TSAs, SMOC1 was rarely methylated in sessile serrated lesions (SSLs), suggesting that SMOC1 methylation may be a diagnostic marker of serrated lesions [12].

In the present study, we aimed to further characterize SMOC1 expression in colorectal tumors. To that end, we performed an immunohistochemical analysis of SMOC1 in a series of colorectal lesions, including serrated lesions, conventional adenomas and CRCs.

## Methods

### Study population

Specimens of colorectal tumors (*n *= 199) and adjacent normal colorectal tissues (*n* = 112) were collected from 173 Japanese patients who underwent endoscopic or surgical resection at Teine-Keijinkai Hospital between 2016 and 2020. Informed consent was obtained from all patients before collection of the specimens. Approval of this study was obtained from the Institutional Review Boards of Teine-Keijinkai Hospital and Sapporo Medical University.

### Endoscopic evaluation and histological analysis

Colorectal tumors were observed at high magnification using high-resolution magnifying endoscopes (CF-HQ290ZI or PCF-H290ZI; Olympus, Tokyo, Japan) after staining with indigo carmine dye and 0.05% crystal violet. Tumors were then treated through endoscopic mucosal resection, endoscopic submucosal dissection or surgical resection. Histological analyses were performed based on the criteria of the World Health Organization (WHO) classification of tumours of the digestive system, 5th edition. Hematoxylin–eosin (HE)-stained sections were available and retrieved for all cases. All specimens were reviewed by pathologists (AT and MO) blinded to the endoscopic diagnosis. Conventional adenomas were subcategorized as non-advanced or advanced adenomas. Advanced adenomas were defined as being 1 cm or more in diameter and/or containing villous components and/or exhibiting high grade dysplasia (HGD).

### Immunohistochemistry and scoring

Immunohistochemical staining was performed as described previously [12]. A rabbit anti-SMOC1 polyclonal antibody (1:1000 dilution, C-20; Sigma-Aldrich) was used. The intensity of SMOC1 staining was graded as strong (3), moderate (2), weak (1) or negative (0). The proportions of positively stained tumor cells were assigned a value of 0 to 10.

Because neoplasm heterogeneity caused varying degrees of immunoreactivity in the slides, we used the sum of each intensity × proportion as an immunohistochemistry (IHC) score (e.g., intensity × proportion = (3) × 5 + (2) × 3 + (1) × 1 + (0) × 1 = IHC score 22; maximum score = 30) to improve accuracy. When TSAs had both flat and protruding components, SMOC1 expression was evaluated in the protruding component. Thereafter, levels of SMOC1 expression were compared between flat and protruding components in order to evaluate its involvement in the progression of TSAs. All slides were independently evaluated by pathologists (AT and MO) who were blinded to the clinical data.

### Statistical analysis

To compare differences in continuous variables between groups, t tests or ANOVA with post hoc Tukey’s tests were performed. Fisher’s exact test or chi-squared test was used for analysis of categorical data. Values of *P* < 0.05 (two-sided) were considered statistically significant. Statistical analyses were carried out using GraphPad Prism ver. 5.0.2 (GraphPad Software, La Jolla, CA, USA).

## Results

### Characteristics of the colorectal tumors in this study

The clinicopathological characteristics of the patients enrolled in this study are summarized in Table 1. The clinicopathological characteristics of each tumor type based on histological findings are summarized in Supplementary Table 1. The majority of SSLs were located in the proximal colon and displayed a flat morphology. By contrast, the majority of TSAs were located in the distal colon and had a protruding morphology (Supplementary Table 1).

**Table 1:** Clinicopathological features of the patients enrolled in this study

Age (y, mean ± SD)		66.4 ± 11.7
Gender	Female	80
	Male	119
Location	Proximal	105
	Distal	94
Size (mm, mean ± SD)		11.3 ± 6.4
Morphology	Depressed	4
	Flat	83
	Flat plus protruding	43
	Protruding	69
Histology	HP	26
	SSL	50
	SSLD/SSL with EIC	14
	TSA	51
	TSA with HGD/EIC	3
	Non-advanced adenoma	17
	Advanced adenoma	30
	EIC	8

*HP* hyperplastic polyp, *SSL* sessile serrated lesion, *SSLD* SSL with dysplasia, *EIC* early invasive cancer, *TSA* traditional serrated adenoma, *HGD* high grade dysplasia

### Analysis of SMOC1 expression in colorectal tumors and normal colonic tissue

**Fig. 1:**
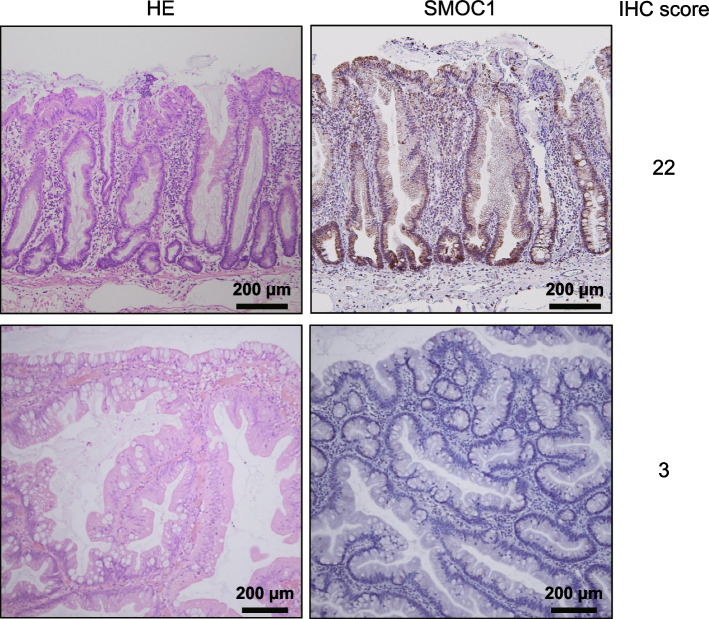
Representative results showing immunohistochemical staining of SMOC1 in specimens of SSL (upper) and TSA (lower). IHC scores are indicated on the right

**Fig. 2:**
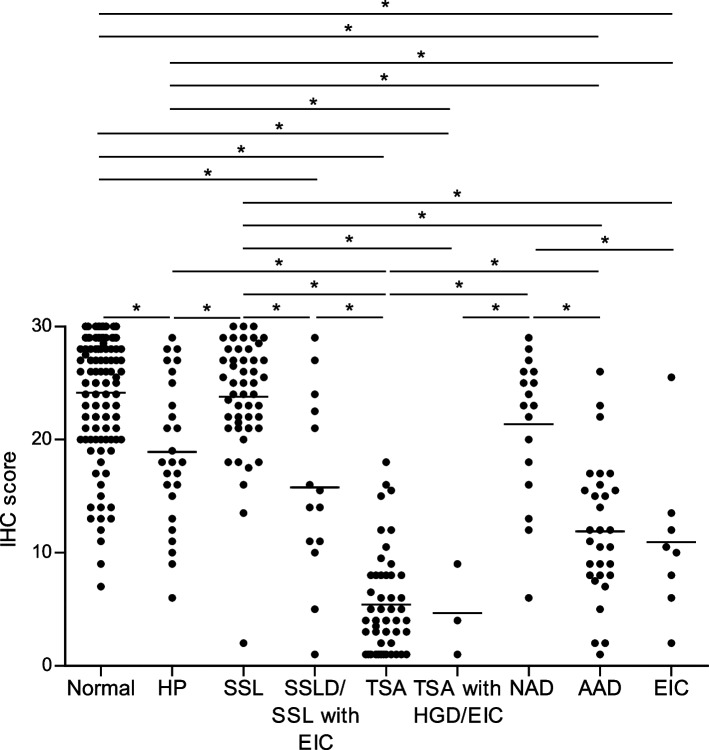
IHC scores summarizing expression of SMOC1 in colorectal tumors (*n* = 199) and normal colonic tissues (*n* = 112). HP, hyperplastic polyp; SSLD, SSL with dysplasia; EIC, early invasive cancer; HGD, high grade dysplasia; NAD, non-advanced adenoma; AAD, advanced adenoma. Tukey–Kramer method, * *P* < 0.05

Representative results showing immunohistochemical staining of SMOC1 in colorectal tumors are shown in Fig. 1. Levels of SMOC1 expression in colorectal tumors and adjacent normal colonic tissues are summarized in Fig. 2. Mean IHC scores in normal colonic tissues and tumors were as follows: normal colon, 24.2; hyperplastic polyp (HP), 18.9; SSL, 23.8; SSL with dysplasia (SSLD)/SSL with early invasive cancer (EIC), 15.8; TSA, 5.4; TSA with HGD/EIC, 4.7; non-advanced adenoma, 21.4; advanced adenoma, 11.9; EIC, 10.9. These results suggest that SMOC1 is abundantly expressed in normal colon, hyperplastic polyps, SSLs and non-advanced adenomas, whereas it is significantly downregulated in TSAs, advanced adenomas and EICs. These results are consistent with our earlier observation that SMOC1 is frequently methylated in TSAs, advanced adenomas and colorectal cancers but is rarely methylated in HPs and SSLs [12]. Notably, SSLDs or SSLs with EIC showed lower levels of SMOC1 expression than SSLs, indicating that downregulation of SMOC1 may be associated with the malignant progression of SSLs.

### SMOC1 expression and clinicopathological characteristics in colorectal tumors

Associations between SMOC1 expression and clinicopathological characteristics in colorectal tumors are summarized in Table 2. Higher levels of SMOC1 expression within tumors correlated positively with proximal colon location and flat tumoral morphology. These results reflected the preferential proximal colon locations and flat morphology of SSLs, where SMOC1 was abundantly expressed.

**Table 2:** Associations between SMOC1 expression and clinical features in colorectal tumors

		IHC score (mean ± SD)	*P*
Age	< 66	15.0 ± 9.2	NS*
	≥ 66	15.4 ± 9.1	
Gender	Female	16.2 ± 9.4	NS*
	Male	14.3 ± 8.9	
Location	Proximal	18.7 ± 8.2	< 0.001*
	Distal	11.0 ± 8.4	
Size (mm)	< 11	15.2 ± 9.2	NS*
	≥ 11	15.1 ± 9.0	
Morphology	Depressed	6.6 ± 3.4	< 0.001^†^
	Flat	22.3 ± 6.0	
	Flat plus protruding	10.0 ± 7.5	
	Protruding	10.1 ± 7.4	

*NS* not significant

^*^Unpaired t-test

^†^Tukey–Kramer method

To further characterize SMOC1 expression in colorectal lesions, we next analyzed associations between SMOC1 expression and clinicopathological characteristics in respective tumor types. Among SSLs, higher levels of SMOC1 expression correlated positively with female gender and flat morphology (Table 3). Interestingly, SMOC1 expression levels did not correlate with tumor locations among SSLs, suggesting that SSLs express high levels of SMOC1 irrespective of the locations (Table 3). A positive correlation between SMOC1 expression and flat morphology was also observed in advanced adenomas and early invasive cancers (Supplementary Table 2). In contrast, no significant correlation between SMOC1 expression and clinicopathological findings was observed in HPs, TSAs, SSLD/SSL with EIC and non-advanced adenomas (Table 3).

**Table 3:** Associations between SMOC1 expression and clinical features in each tumor type

		IHC score in HP (mean ± SD)	*P*	IHC score in SSL (mean ± SD)	*P*	IHC score in TSA (mean ± SD)	*P*
Age	< 66	18.4 ± 5.7	NS*	24.1 ± 4.1	NS*	4.5 ± 4.2	NS*
	≥ 66	19.5 ± 7.4		23.5 ± 5.9		6.4 ± 4.5	
Gender	Female	19.5 ± 6.7	NS*	25.4 ± 3.4	< 0.01*	5.5 ± 3.6	NS*
	Male	18.8 ± 6.6		21.7 ± 6.1		5.3 ± 5.0	
Location	Proximal	17.8 ± 6.0	NS*	23.6 ± 5.1	NS*	6.3 ± 6.1	NS*
	Distal	20.8 ± 7.1		26.2 ± 4.1		5.2 ± 4.0	
Size (mm)	< 11	19.0 ± 6.8	NS*	23.1 ± 4.2	NS*	5.9 ± 4.7	NS*
	≥ 11	18.0 ± 0		24.3 ± 5.7		4.1 ± 2.9	
Morphology	Flat	18.7 ± 6.4	NS^†^	24.5 ± 4.1	< 0.05^†^		NS*
	Flat plus protruding	17.0 ± 1.4		16.7 ± 12.7		5.8 ± 4.5	
	Protruding	20.3 ± 8.1		21.0 ± 3.0		5.0 ± 4.3	

*HP* hyperplastic polyp, *SSL* sessile serrated lesion, *TSA* traditional serrated adenoma, *NS* not significant

^*^Unpaired t-test

^†^Tukey–Kramer method

**Fig. 3:**
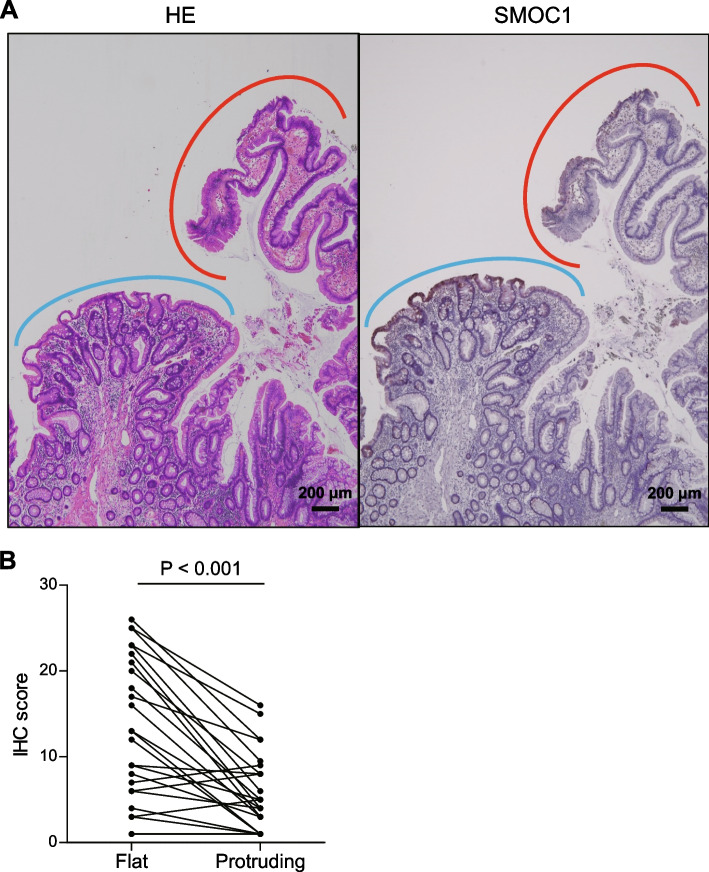
SMOC1 expression in TSAs containing both flat and protruding components. **A** Immunohistochemical staining of SMOC1 in a representative TSA specimen containing flat (blue) and protruding (red) components. **B** IHC scores summarizing expression of SMOC1 in TSAs containing both flat and protruding components (*n* = 25)

We previously observed that SMOC1 is frequently methylated in TSAs with protruding morphology [12]. We therefore assessed SMOC1 expression levels in TSAs containing both flat and protruding components (Fig. 3A). We found that the protruding components showed significantly lower levels of SMOC1 expression than the flat components, suggesting that silencing SMOC1 may be associated with the progression of TSAs (Fig. 3B).

## Discussion

In this study, we showed that reduced expression of SMOC1 is associated with progression of colorectal tumors, including TSAs and conventional adenomas. Notably, levels of SMOC1 expression strikingly differed among serrated lesion subtypes; whereas SMOC1 was abundantly expressed in HPs and SSLs, it was significantly downregulated in TSAs. These results are consistent with our earlier finding that aberrant DNA methylation of SMOC1 is associated with progression of TSAs and conventional adenomas [12]. Our finding that SSLDs and SSLs with EIC exhibit lower levels of SMOC1 expression than SSL also suggest that downregulation of SMOC1 may be associated with malignant progression of SSLs.

SMOC1 is a member of the SPARC (secreted protein acidic and rich in cysteine) family and was first identified as a secreted modular calcium-binding glycoprotein widely expressed in various tissues [13]. SMOC1 is localized in the basement membrane of cells, where it plays a role in integrin-matrix interactions and cell adhesion [13, 14]. Although the biological function of SMOC1 is not fully understood, multiple studies have reported its involvement in development. In Xenopus, for example, SMOC1 antagonizes bone morphogenetic protein (BMP) signaling and is essential for postgastrulation development [15]. In humans, SMOC1 reportedly acts as a putative regulator of osteoblast differentiation [16]. SMOC1 is also essential for ocular and limb development in humans and mice, and SMOC1 mutations were found to cause Waardenburg Anophthalmia syndrome [17–19]. Another study showed that SMOC1 produced by endothelial cells promotes angiogenesis through regulation of transforming growth factor (TGF)-β signaling [20]. Moreover, a recent study revealed that SMOC1 is a glucose-responsive hepatokine that regulates glucose homeostasis [21]. Together, these studies demonstrate that SMOC1 is a multifunctional protein that modulates cell–matrix interactions and various cellular signaling pathways.

SMOC1 has also been implicated in tumorigenesis. In brain tumors, SMOC1 interacts with tenascin-C, an extracellular matrix protein overexpressed in various tumor types [22]. In addition, multiple lines of evidence suggest that SMOC1 is associated with the prognosis of gliomas. For instance, using a dataset from The Cancer Genome Atlas (TCGA), Zhang et al. searched for differentially expressed genes in gliomas and identified a seven-gene signature associated with the disease [23]. Among these genes, higher expression of SMOC1 correlated positively with a better prognosis in glioma patients. Wang et al. also reported that SMOC1 was upregulated in low-grade gliomas (LGGs) and that higher SMOC1 expression correlated with a better prognosis in LGG patients [24]. Interestingly, SMOC1 expression correlated negatively with infiltration by B cells, CD4 + T cells, CD8 + T cells, macrophages and dendritic cells in LGGs, suggesting that SMOC1 may be associated with the tumor microenvironment of glioma [24]. In another recent study, a series of basement membrane-related genes, including SMOC1, was used to establish a risk prediction model for gliomas [25]. Consistent with these reports, hypermethylation of SMOC1 is reportedly associated with shorter survival of glioma patients [26]. In contrast to gliomas, positive expression of SMOC1 was reportedly associated with recurrence and a poorer prognosis in pancreatic neuroendocrine tumors [27]. These studies suggest that dysregulation of SMOC1 is involved in tumorigenesis in multiple organs, though its contribution to malignant progression may differ among tumor types.

Although there have been few studies focused on SMOC1 in colorectal tumors, recent multi-omics analyses suggest the involvement of SMOC1 in CRCs. For instance, Huang et al. identified differentially expressed long noncoding RNAs, microRNAs and messenger RNAs in colon cancers in TCGA datasets and assessed their association with patient survival [28]. They found that SMOC1 expression is associated with a better prognosis in colon cancer patients, suggesting a protective role for SMOC1. In addition, Lie et al. used omics data from more than 6000 CRC patients to categorize CRCs into novel molecular categories termed gene interaction perturbation network-based subtypes (GINS) [29]. Among them, GINS3 was characterized by high tumor purity, immune-desert, activation of EGFR and ephrin receptors, chromosomal instability, fewer KRAS mutations, SMOC1 methylation, immunotherapeutic resistance, and high sensitivity to cetuximab and bevacizumab. These results are consistent with SMOC1 playing a tumor suppressor role and suggest that silencing of SMOC1 may be involved in the development of CRC.

## Conclusion

In summary, we showed here that downregulation of SMOC1 is associated with the progression of precancerous colorectal lesions. Our findings suggest that expression of SMOC1 may be a diagnostic marker of serrated lesions as well as a predictive marker of colorectal tumors at high risk of developing into cancer.

### Supplementary Information

**Supplementary Material 1.**

## Data Availability

The datasets generated during and/or analyzed during the current study are available from the corresponding author on reasonable request.

## Abbreviations

SMOC1: SPARC-related molecular calcium-binding 1
SPARC: Secreted protein acidic and rich in cysteine
TSA: Traditional serrated adenoma
CRC: Colorectal cancer
SSL: Sessile serrated lesion
SSLD: Sessile serrated lesion with dysplasia
HP: Hyperplastic polyp
EIC: Early invasive cancer
HGD: High grade dysplasia
NAD: Non-advanced adenoma
AAD: Advanced adenoma
CIMP: CpG island methylator phenotype
HE: Hematoxylin–eosin
IHC: Immunohistochemistry
