# Supplementary material for: Downregulation of SMOC1 is associated with progression of colorectal traditional serrated adenomas
Source: BMC Gastroenterol. 2024 Mar 1;24:91. doi: 10.1186/s12876-024-03175-1 (PMC10905814; doi:10.1186/s12876-024-03175-1)
Supplement: Supplementary file 1 — Supplementary Material 1. [file 12876_2024_3175_MOESM1_ESM.pdf]

Supplementary Table 1. Clinical findings for each tumor type

		HP	SSL	SSLD/SSL with EIC	TSA	TSA with HGD/EIC	Non-advanced adenoma	Advanced adenoma	EIC
		n=26	n=50	n=14	n=51	n=3	n=17	n=30	n=8
Age (y, mean ± SD)		66.2 ± 11.5	66.1 ± 9.9	69.8 ± 13.0	65.6 ± 12.3	53.7 ± 13.5	72.5 ± 7.9	65.2 ± 13.4	63.4 ± 12.7
Gender	Female	6	28	9	22	1	4	8	2
	Male	20	22	5	29	2	13	22	6
Location	Proximal	16	47	12	9	0	10	7	4
	Distal	10	3	2	42	3	7	23	4
Size (mm, mean ± SD)		7.2 ± 3.3	12.8 ± 5.3	13.2 ± 4.0	10.1 ± 5.9	24.0 ± 18.2	5.2 ± 1.8	13.6 ± 3.9	19.0 ± 10.8
Morphology	Depressed	0	0	0	0	1	0	0	4
	Flat	18	44	3	0	0	12	2	3
	Flat plus protoruding	2	3	10	25	2	0	3	0
	Protoruding	6	3	1	26	0	5	25	1

HP, hyperplastic polyp ; SSL, sessile serrated lesion; SSLD, SSL with dysplasia; EIC, early invasive cancer; TSA, traditional serrated adenoma; HGD, high grade dysplasia

Supplementary Table 2. Associations between SMOC1 expression and clinical features in each tumor type

		IHC score in SSLD/SSL with EIC (mean ± SD)	P	IHC score in NAD (mean ± SD)	P	IHC score in AAD/EIC (mean ± SD)	P
Age	<66	18.7 ± 6.8	NS*	24.0 ± 1.4	NS*	12.6 ± 6.4	NS*
	≥66	15.0 ± 8.6		21.0 ± 6.7		11.0 ± 5.9	
Gender	Female	15.5 ± 7.5	NS*	19.0 ± 6.1	NS*	12.2 ± 6.1	NS*
	Male	16.3 ± 10.2		22.1 ± 6.5		11.5 ± 6.2	
Location	Proximal	15.5 ± 8.7	NS*	21.0 ± 6.2	NS*	10.9 ± 6.1	NS*
	Distal	17.5 ± 4.9		21.9 ± 7.2		12.0 ± 6.2	
Size (mm)	<11	15.4 ± 10.5	NS*	21.4 ± 6.4	NA	14.8 ± 8.8	NS*
	≥11	16.0 ± 7.6				10.7 ± 4.8	
Morphology	Depressed		NA		NS*	6.6 ± 3.6	<0.05†
	Flat	22.5 ± 9.2		23.0 ± 6.0		17.8 ± 6.4	
	Flat plus protruding	15.9 ± 6.8				11.8 ± 5.3	
	Protruding	1.0 ± 0		16.0 ± 5.0		11.3 ± 5.8	

SSLD, SSL with dysplasia; EIC, early invasive cancer; NAD, non-advanced adenoma; AAD, advanced adenoma; NS, not significant; NA, not available
*Unpaired t-test, †Tukey-Kramer method
